# Evaluating the implementation of the 13-valent pneumococcal vaccine supplementary dose program in Australian primary health care settings

**DOI:** 10.1186/s12913-015-0738-y

**Published:** 2015-03-18

**Authors:** Kirsten F Ward, Marianne Trent, Brynley P Hull, Helen E Quinn, Aditi Dey, Robert I Menzies

**Affiliations:** National Centre for Immunisation Research and Surveillance of Vaccine Preventable Diseases (NCIRS), The Children’s Hospital at Westmead, Cnr Hawkesbury Road and Hainsworth Street, Westmead, NSW 2145 Australia; North Coast Public Health Unit, 31 Uralba Street, Lismore, NSW 2480 Australia; Discipline of Paediatrics and Child Health, University of Sydney, Cnr Hawkesbury Road and Hainsworth Street, Westmead, NSW 2145 Australia

**Keywords:** Immunization, Pneumococcal, Evaluation, Vaccine

## Abstract

**Background:**

The availability of new pneumococcal conjugate vaccines covering a broader range of serotypes, has seen many countries introduce these into their national immunisation program. When transitioning from 7-valent to 13-valent pneumococcal conjugate vaccines, Australia is one of a small number of countries that included a supplementary dose of the 13-valent pneumococcal conjugate vaccine to offer protection against additional serotypes to an expanded age group of children. An evaluation of the implementation and uptake of the 13-valent pneumococcal conjugate vaccine supplementary dose was undertaken in two local health districts (LHDs) in New South Wales, Australia.

**Methods:**

A self-administered postal survey of immunisation providers in the Northern New South Wales and Mid North Coast LHDs. Trends in vaccine ordering were examined. Coverage was assessed using data from the Australian Childhood Immunisation Register (ACIR).

**Results:**

Of the 177 surveys sent, 125 were returned (70%). Almost all providers (96%) were aware of the 13vPCV supplementary dose program though took an opportunistic approach to program promotion and parental reminders. Supplementary doses of 13vPCV were ordered for 37% of the eligible cohort, mostly in the program’s first six months. Coverage as recorded on the ACIR was 27%, though was lower in older children and those not due for scheduled childhood vaccines. Of the children who received the 13vPCV supplementary dose, 3% received it at the same time as vaccines due at 12-months of age, and 44% at the time of those due at 18-months of age.

**Conclusion:**

Despite the high awareness of the program, reported coverage was lower than that for other PCV supplementary dose programs in Australia and internationally. This may be influenced by providers’ largely opportunistic approach to implementation, under-reporting to the ACIR or vaccine uptake. Lessons learned from this evaluation are relevant for future time-limited childhood vaccination programs. Prior to commencement, providers should be informed about the importance of catch-up/supplementary vaccination for their patients and their active role in promoting this. They should also receive program information before parents. An understanding of parental reasons for non-receipt of time-limited childhood vaccines and evaluation of the effect of aligning supplementary (or catch up) vaccination programs with the NIP schedule would be useful to inform future programs.

## Background

In the last decade, vaccines have been introduced to Australia’s National Immunisation Program (NIP) to protect children against five additional diseases [1]. In order to maximise protection for high-risk groups and to control disease at a population level, time-limited funding for a ‘catch-up’ series/dose or a ‘supplementary dose’ of newly introduced vaccines is often provided for additional cohorts. These programs have predominantly targeted school-aged children and adolescents [2-5].

When 7-valent pneumococcal conjugate vaccine (7vPCV) was introduced into Australia’s NIP for infants in 2005, the Australian Government funded a 1-year catch-up program in that year for children 2–23 months of age. The number of doses required for catch-up varied depending on the age at which the first dose had been given [6,7]. From July 2011, the 13-valent pneumococcal conjugate vaccine (13vPCV) replaced 7vPCV in all states/territories except the Northern Territory, where the 13vPCV replaced the 10-valent pneumococcal conjugate vaccine (10vPCV) [8]. From October 2011 to September 2012 the Australian Government funded a single supplementary dose of 13vPCV for children aged 12–35 months (1–3 years) who had not received a dose of this vaccine or the 10vPCV in their primary course [9]. This was felt to be a sufficiently cost-effective measure to provide these children, who are at high risk of disease, the benefit of protection from the additional six serotypes covered by 13vPCV [10]. The NIP schedule for 13PCV is 3 + 0 for non-Indigenous children and 3 + 1 for Indigenous children in high risk states [7].

Currently, 13vPCV is approved for use in over 120 countries, 60 of which offer it as part of national or regional immunisation programs. [11] However, only a minority have offered a supplementary dose of 13vPCV, mostly for children up to five years of age. These include the United States (vaccine not nationally funded or time-limited), Italy (vaccine funded and not time limited) and two Canadian provinces, Manitoba (vaccine funded and not time limited) and Ontario (vaccine funded for 2011 only) [12-15]. In light of this, there is considerable national and international interest in the unique 13vPCV supplementary dose program in Australia.

Evaluations of time-limited catch-up or supplementary vaccination programs targeting infants, either in Australia or internationally, are limited and predominantly focus on measuring vaccine coverage and the impact on disease. Notwithstanding the importance of such evidence, effective implementation is also a significant factor influencing the success of these programs. [16,17] This evaluation of the 13vPCV supplementary dose program in the Northern New South Wales (NNSW) and Mid North Coast (MNC) Local Health Districts (LHDs) aimed to understand a) provider awareness and approaches to implementation, and b) vaccine coverage, along with accuracy and reliability of these estimates.

## Methods

### Provider survey

A self-administered postal survey of all immunisation providers in the NNSW and MNC LHDs was undertaken to determine awareness of the 13vPCV supplementary dose program and approaches to implementation. Immunisation providers were defined as general practices (GP), Aboriginal Medical Services (AMS) or community health centres (CH) with a NSW Ministry of Health vaccine account number (VAN) and listed on public health unit (PHU) records. These “immunisation providers” represented 93% (177/183) of all GP, AMS and CH centres in these LHDs.

In-depth knowledge of the local immunisation coordinator, together with previous Australian NIP evaluations [18], informed the development of the survey, which was refined following piloting with three immunisation providers outside of the NNSW and MNC LHDs. The 17-question survey had a combination of open and closed questions about program information received, resources used, recall/reminder activities and notification practices to the Australian Immunisation Register (ACIR). Surveys were accompanied by a one-page cover letter, signed by the Director of the North Coast PHU. They were to be completed at the organisational level by the individual(s) with primary responsibility for immunisation services. Surveys were posted in March 2012 with an initial 2-week deadline for response. Non-respondents were individually faxed a second copy 4 weeks following the initial deadline, with end-June 2012 as the final response cut-off.

The Northern New South Wales Medicare Local and the North Coast PHU promoted the survey via advertisements in routine newsletters and weekly faxes, emails to lists of general practice nurses (PNs) and personal reminders at visits to GPs/AMSs.

Completed surveys were scanned into Cardiff Teleforms v10.5.2® [19], which aggregated responses for output to Microsoft Excel for data cleaning. The surveys were de-identified prior to scanning and data cleaning. Recoding and statistical analysis were performed using SAS Enterprise Guide v4.3 [20]. The sample was weighted by LHD and provider type (GP, AMS, CH). Where appropriate, response variables were collapsed into dichotomous variables (‘yes’ or ‘no’). For questions that used a Likert scale [21], ratings were calculated as a percentage of those who responded to each question. Content analysis [22] was used to generate key themes from responses to open-ended questions. Point estimates and 95% confidence intervals (CI) were calculated where appropriate, with an odds ratio calculated and chi square test used when comparing dichotomous variables. A p value <0.05 was considered statistically significant.

In addition to the survey, the Medicare Local and PHU provided detailed records of the type and date of education/promotion activities undertaken in relation to the 13vPCV supplementary dose program. Descriptive analysis was performed on these data to determine frequency of activity type (e.g. newsletter, face-to-face visit) and month of implementation. Also, to determine generalisability of the survey findings, the Rural, Remote, Metropolitan Area classification (RRMA) [23] distribution of providers in NNSW and MNC LHDs was compared with that of NSW as a whole. The NSW distribution was obtained from the 2012 Annual Survey of Divisions of General Practice (DGP) [24], which classifies DGPs by RRMA. This classification was applied to postcodes of AMSs [25] and CH centres [26] in NSW, both obtained from publicly available data.

### Vaccine orders

The NSW Ministry of Health (MOH) provided line-listings of PCV monthly orders placed by immunisation providers with a VAN in the NNSW and MNC LHDs. These covered the period from October 2010, 12 months prior to commencement of the supplementary dose program, to December 2012, the end of evaluation period and three months after the program end-date. It was not possible to separate orders of 7vPCV and 13vPCV or those for the supplementary dose program vs the routine NIP schedule. The North Coast PHU supplied the total number of PCV doses reported as wasted per month, from May 2011 to December 2012. The median of the available wastage data was deducted from the raw number of doses ordered each month. The monthly orders (less wastage) for the 12 months before and three months after the 13vPCV supplementary dose program were then averaged to give the ‘baseline’ ordering rate (doses) per month. The number of additional doses ordered per month during the supplementary dose program was estimated by deducting the baseline ordering rate from the number of doses ordered for each month of the program. Monthly totals of ordered doses (excluding wastage) were graphed to examine ordering trends prior, during and after the 13vPCV supplementary dose program.

PHU records of live births by LHD were used to determine the number of eligible children, by month, for the 13vPCV supplementary dose program (born 1 October 2008 to 31 December 2010).

### Vaccination coverage

Vaccination coverage was assessed using data from the ACIR as at 30 December 2012 [27]. Receipt of a supplementary dose of 13vPCV was assessed for children born 1 October 2008 to 31 December 2010 (aged 12–35 months) as children in this cohort were not scheduled to receive a dose of 13vPCV in their primary course of PCV. Receipt of a supplementary dose of 13vPCV was defined as a fourth dose of 13vPCV recorded on the ACIR with an administration date during the program period. In addition, the historical average number of 13vPCV doses reported to the ACIR per month was then subtracted from the number of doses reported each month during the supplementary dose program period. The remaining doses were assumed to be mis-coded supplementary doses of 13vPCV and added into coverage estimates. Due to coding limitations it was not possible to exclude booster doses of 13vPCV for medically at-risk children or to capture doses recorded under generic codes (‘other vaccine’).

Receipt of another vaccine on the same day as the 13vPCV supplementary dose was assessed by matching administration dates for eligible children during the program period. The method of notification to the ACIR for reported 13vPCV supplementary doses was obtained for the three provider types (GPs, AMSs and CHs) from ACIR data as at 31 December 2012.

The North Coast Area Health Service Human Research Ethics Committee approved this evaluation as a quality assurance activity. It was conducted without financial or other support from any vaccine manufacturing company or individual or entity acting on behalf of the vaccine manufacturing industry.

## Results

Of the 177 surveys sent to immunisation providers, 125 were returned completed (70% response rate). Of the remaining 52 non-returned surveys, four were not returned due to service closure/amalgamation (4/52, 7%), five were returned incomplete (5/52, 10%) and 43 were not returned at all (43/52, 83%). The majority of non-responders were GPs (40/52, 77%), most of whom had not ordered 13vPCV during the survey period despite ordering other NIP vaccines during this time. Response rates did not differ significantly between the LHDs, though were higher amongst AMSs (10/13, 77%) and CH centres (19/20, 95%) than GPs (96/144, 67%).

All providers in the survey sample were from the RRMA ‘rural zone’ (R1–3) [23]. The distribution of provider types in the sample was similar to that of all rural NSW, with a slightly higher proportion of AMSs (8% vs 2%, p < 0.0001) and community health centres (15% vs 11%, p = 0.009). GPs in the sample were more likely than those in NSW to be large (≥6 doctors, 35% vs 15%, p < 0.0001) (Table 1). GPs were the predominant immunisation provider in the sample as well as in rural and all NSW. Immunisation-authorised registered nurses (RNs) were the most common health professional administering vaccines across all provider types (70/125, 56%).

**Table 1:** **Comparison of attributes of the survey sample with NSW and rural NSW**

	**Survey samplen (%)**	**NSW n (%)**	**NSW RRMA 1–3 n (%)**
General practice	96 (77)^a^	2712 (87)	393 (71)
Aboriginal Medical Service (AMS)	10 (8)^a,b^	52 (2)	28 (5)
Community Health	19 (15)^a^	347 (11)	130 (24)
Solo general practice	14 (15)^a,b^	1198 (44)	154 (39)
General practice with 2–5 doctors	42 (44)	1117 (41)	188 (48)
General practice with ≥6 doctors	34 (35)^a,b^	397 (15)	51 (13)
General practice with practice nurse	57 (59)^a,b^	1206 (45)	310 (79)

Footnotes

^a^Statistically significant difference (p < 0.05) between our sample and all NSW providers.

^b^Statistically significant difference (p < 0.05) between our sample and all providers in NSW RRMA 1–3.

Most respondents stored patient information only electronically (86/125, 69%), though a considerable number also used a mix of paper and electronic records (25/125, 20%) with community health centres being almost solely paper-based. Most (101/125, 81%) respondents used electronic patient management software, predominantly Medical Director® (61/101, 60%) or Best Practice® (24/101, 24%).

### Awareness

Overall, 96% of respondents were aware of the 13vPCV supplementary dose program (weighted n = 120), with no significant difference by LHD or provider type. The majority of respondents were made aware of the program through up to three sources. Of those that found out about the program from a single source (n = 22), the most common were the letter from the Australian Government Chief Medical Officer (CMO) to providers (16/22, 57%) and the Medicare Local newsletter (6/22, 20%); combined, these accounted for half (18/34, 51%) of the dual sources of awareness about the program. Other pre-defined sources of provider awareness about the program included the NSW Ministry of Health (MOH) website (19/125, 15%), information accompanying the vaccine delivery (41/125, 33%), the initial CMO letter to parents of eligible children sent in September 2011 (27/125, 22%), and face-to-face meetings held by the Medicare Local (42/125, 34%). No respondents became aware of the program through social media. For a GP, having a practice nurse (PN) did not significantly influence awareness of the program, as overall awareness was high.

### Resources and promotion

The survey examined the use of three specific resources to support implementation of this program: Australian Government provider guidelines [28], NSW MOH ‘Pneumococcal for Children’ fact sheet, and a resource pack from the vaccine supplier (Pfizer Australia Pty Ltd). The latter included a product information card, a supplementary dose program handbook for health professionals, a pad of patient information leaflets, a magnet and recall letter template (electronic file). Just under half of respondents used only one of these resources to implement the program (59/125, 47%), most commonly the Australian Government Provider Guidelines (44/59, 75%) or the MOH fact sheet (8/59, 14%).

Of the providers who became aware of the program from NSW MOH (via their website or information sent with vaccine orders, n = 50), more than half (28/50, 56%) reported that they did not use the MOH fact sheet. In contrast, if providers found out about the program via the CMO letter posted to providers they were more likely to use the Australian Government provider guidelines that accompanied the letter (OR 2.5, 95% CI: 1.07–5.9, p < 0.05) than those who found out about the program from other sources. When examining attitudes to information provided about the program, most respondents agreed the information they received was sufficient, easy to access and clear (Table 2).

**Table 2:** **Perspectives about qualities of the information received on the 13vPCV supplementary dose program from survey respondents (n = 120)**

	**Agree n (%)**	**Neutral n (%)**	**Disagree n (%)**
Timely	93 (77)	7 (6)	21 (17)
Sufficient	102 (86)	8 (7)	8 (7)
Easy to access	101 (87)	11 (10)	4 (3)
Clear	109 (93)	5 (4)	4 (3)

One-third (42/125, 34%) of respondents did not actively promote the 13vPCV supplementary dose program to patients. Of the two-thirds of providers who did promote the program, most used one (22/83, 27%) or two (27/83, 33%) promotional resources. Brochures (12/35, 34%) and posters (9/35, 26%) were the most commonly used single resources. These resources were the most commonly used combination of resources (31/42, 74%). The Medicare Local and North Coast PHU undertook a variety of education and promotional activities that were predominantly implemented during the first two months of the program (October 2011 = 15% and November 2011 = 39%) and the second last month of the program (August 2012 = 15%). The Medicare Local included information about the program in monthly newsletters, group emails to health professionals and weekly faxes, all of which primarily targeted AMSs and GPs. Some Medicare Local staff also provided verbal information about the program at face-to-face group education sessions (n = 11) and routine visits to GPs and AMSs. Representatives of the vaccine supplier also visited GPs and AMSs and provided their 13vPCV resource pack.

### Reminders

Most respondents indicated they regularly sent reminders for routine childhood vaccination (116/125, 93%) although over three-quarters (96/125, 78%) did not send written reminders specifically for the 13vPCV supplementary dose program. This was mainly as they felt parents already knew about the program from the CMO letter or were told about it when they visited an immunisation provider for routine childhood vaccination for an eligible child or siblings. Lack of time and staff and insufficient systems for generating reminders also influenced providers’ decisions not to send reminders for this program.

*Not enough available staff to provide this reminder service; not really necessary as most parents have received a letter and they [eligible children] are all offered supplementary dose of Prevenar13® on presentation to the clinic*. (general practice)

Of the written parental reminders sent, all were from GPs and were predominantly coordinated by the PN (18/27, 66%). Reminders were mostly provided via a mailed letter/postcard (5/27, 18%), telephone (6/27, 21%) or a combination of both (2/27, 7%). No provider reported using short messaging services (SMS) or email to send reminders for the 13vPCV supplementary dose program. Irrespective of sending written reminders, the majority of providers verbally informed parents about this program at either the 18-month vaccination visit (61/125, 49%) or when an eligible child presented for any other health condition (3/125, 2%), or at both these opportunities (28/125, 22%).

Parents of eligible children who had not had a supplementary dose of 13vPCV recorded on the ACIR (as at 7 August 2012) received a personal reminder letter from the CMO in late August 2012. Due to the timing of the provider survey, provider awareness and experiences following dissemination of this letter could not be examined as part of this evaluation.

### Vaccine orders

**Figure 1:**
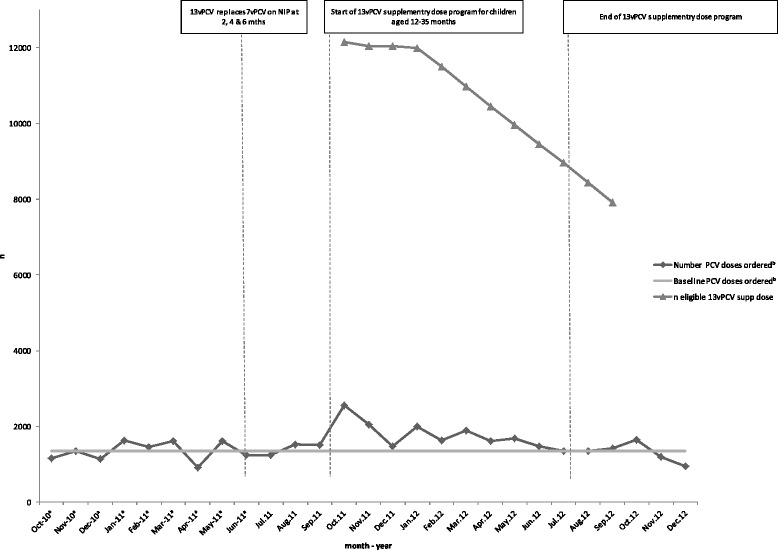
**Eligible cohort for the 13vPCV supplementary dose and ordering of PCV in the Northern New South Wales and Mid North Coast Local Health Districts by month, October 2010 to December 2012.**

Ordering trends for all PCVs in the NNSW and MNC LHDs are presented in Figure 1. The baseline rate of PCV ordering for the routine 2, 4 and 6-month doses was 1346 doses per month (excluding wastage). It was estimated that 4768 additional doses (excluding wastage) were ordered throughout the 12 months of the program, covering 37% of the eligible cohort (12,981/35,083). Almost half the additional doses (2146/4768, 45%) were ordered in the first quarter of the program (by end-December 2011) with the majority (3719/4768, 78%) ordered by half way through the program (end-March 2012). Peaks in ordering were observed in the first month of the program (October 2011), prior to the start of the school year (January 2012) and at the time the survey was distributed (March 2012).

### Reporting supplementary doses

The majority of providers (119/125, 95%) reported that they routinely notified 13vPCV supplementary doses to the ACIR. The most common methods for this, as illustrated by both the ACIR and survey data, were direct transfer from practice software/online claiming (56/119, 47%) or entered directly on the ACIR website (48/125, 40%) (Table 3).

**Table 3:** **Method of notification to the ACIR for 13vPCV supplementary doses from survey respondents (n = 120) and ACIR data at 31 December 2012**

	**Aboriginal Medical Service**		**Community Health**		**General practice**		**All providers**	
	**Survey data** ^**a**^ ***% providers***	**ACIR data** ^**b**^ ***% doses***	**Survey data** ^**a**^ ***% providers***	**ACIR data** ^**b**^ ***% doses***	**Survey data** ^**a**^ ***% providers***	**ACIR data** ^**b**^ ***% doses***	**Survey data** ^**a**^ ***% providers***	**ACIR data** ^**b**^ ***% doses***
Direct transfer from practice management software	40	–	5	2	59	64	47	47
Entered directly onto ACIR secure website	47	100	85	75	27	26	40	39
Paper forms posted to ACIR	13	–	5	23	8	11	8	14
Patient lists faxed to ACIR	–	No data	–	No data	1	No data	1	No data
Telephone direct to ACIR	–	No data	5	No data	5	No data	4	No data

Footnotes

^a^Survey data is the proportion of respondents who indicated they notified supplementary doses of 13vPCV to the ACIR via each notification method.

^b^ACIR data is the proportion of recorded doses of 13vPCV for each provider in the NNSW and MNC LHDs who notified by each method.

A minority of survey respondents reported challenges with reporting or non-reporting to the ACIR (13/125, 10%). The main challenge was the initial lack of a specific field code for 13vPCV on both patient management software and the ACIR forms (electronic and paper). Other reported challenges included lack of awareness or time to report and uncertainty about how to enter the new vaccine correctly.

### Coverage

**Figure 2:**
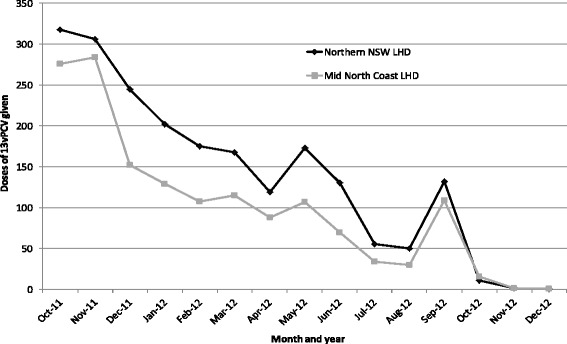
**Coverage as recorded on the ACIR, by month, for the supplementary dose** ^**a**^ **of 13vPCV in children born 1 October 2008 to 31 December 2010 in the Northern New South Wales and Mid North Coast Local Health Districts as at 31 December 2012.** a. ‘Supplementary doses’ reported to the ACIR for eligible children during the 13vPCV supplementary dose program include fourth doses of 13vPCV and fourth dose of 7vPCV minus the baseline number of reported fourth doses of 7vPCV per month (n=1).

Data from the ACIR indicate that 27% of the eligible cohort (children aged 12–35 months between October 2011 and September 2012) in NNSW and MNC LHDs received a supplementary dose of 13vPCV during the 12 months of the program. Uptake was similar in both LHDs (NNSW = 27.2%, MNC = 27.3%) and was lower than the average for all NSW (32.8%, range 27.2–39.0%). Coverage was higher in children 12–23 months of age (36.3%) than those 24–35 months of age (14.8%). Uptake was highest in the first two months of the program, and then declined throughout, with an increase in the final month of the program (Figure 2). Of those children who received the 13vPCV supplementary dose, 3% received it at the same time as vaccines due at 12 months of age (first dose of measles-mumps-rubella vaccine, first dose of meningococcal type C vaccine and fourth dose of *Haemophilus influenzae* type b [Hib] vaccine). Furthermore, 44% of children who received the 13vPCV supplementary dose received it at the same time as the vaccine given at 18 months of age (varicella).

## Discussion

This evaluation found high awareness of the 13vPCV supplementary dose program among immunisation providers in the NNSW and MNC LHDs and providers felt they were provided with sufficient information to support implementation. Most took an opportunistic approach to program promotion and parental reminders. The intensity of education and information dissemination undertaken by the PHU and the Medicare Local decreased over the duration of the program. Vaccines were ordered for 37% of the eligible cohort, with almost all (78%) ordered in the program’s first six months. Coverage as recorded on the ACIR was low (27%), most likely reflecting a combination of under-reporting and low uptake, the latter particularly in older children and those not due for an NIP-scheduled vaccine.

The evaluation was conducted in two rural areas of one Australian state with known lower than average uptake of childhood vaccines and a higher than average proportion of registered conscientious objectors to vaccination (4% vs 2.5% nationally) [29]. Despite this, comparing coverage in the NNSW and MNC LHDs for the previous 7vPCV catch-up program (1–2 doses, 40%) and the 13vPCV supplementary dose program (1 dose, 36%) in a common target group of children (12–23 months of age) illustrates similar uptake in both programs. Given this and the low uptake in the United States supplementary dose program [30], findings related to program implementation from this evaluation are likely to be more broadly generalisable.

### Program awareness and implementation

Only a minority of respondents remained unaware of the 13vPCV supplementary dose program. However, there were no particular characteristics common to these providers highlighted in the available data so their lack of awareness may have simply been a result of the information getting lost in the large volume of material received by primary care providers, particularly GPs [31]. However, the level of awareness about the program does not take into consideration survey non-respondents, most of whom had not ordered 13vPCV vaccine by the end of the survey period, and who were thus likely unaware, or potentially unsupportive, of the 13vPCV supplementary dose program (non-response bias) [32]. Despite this, the overall response rate (70%) to this survey was higher than previous vaccination-related surveys of GPs and PNs in these LHDs (47%) and nationally (32%) [33,34].

Most providers who were aware of the program came to know about it through multiple sources, reflecting the provision of information by multiple stakeholders who support NIP implementation in Australia and highlighting the important role of the Australian Government and Medicare Locals [35] in disseminating such information to providers. High use of the Australian Government provider guidelines [28] was consistent with GPs’ use of these for the National HPV Vaccination Program [33]. Some general practices felt more timely information was required initially, mostly as patients presented with the CMO letter to parents prior to a similar letter reaching providers. Provision of program guidelines from the Australian Government or via the Medicare Local with an accompanying letter to providers about program commencement appeared to enhance the use of these guidelines, in contrast to disseminating program resources separately from initial program information.

Most providers did not send written parental reminders about this program, mainly as they felt the CMO letter to parents was sufficient. Coverage data illustrate increases in uptake at the time CMO letters were sent to parents (September 2011 and August 2012). There is considerable evidence of the effectiveness of personally addressed reminder letters to parents in increasing childhood vaccination uptake [36], particularly for older children for whom vaccination is most delayed [3]. SMS reminders are also an efficient, cost-effective and acceptable reminder mechanism for childhood immunisation, and have the potential to reach low-income families and socially marginalised groups [37-40]. In light of this and the availability and affordability of this technology, mechanisms to enhance use of SMS reminders for time-limited vaccination programs should be further explored.

### Vaccine demand

Ordering data were not able to be separated by vaccine type (7vPCV vs 13vPCV) and were only available by month, thus limiting the assessment of trends over time and the ability to link definitively with program-related activities. Provider-level ordering trends could not be identified due to considerable heterogeneity in ordering patterns. More precise data on vaccine type was not available and tracking of individual doses were not possible, both of which limited the capacity to determine if an ordered dose of 13vPCV was administered as a supplementary dose. Despite these limitations, ordering data were useful to estimate the maximum uptake of the 13vPCV supplementary dose and to monitor demand for the vaccine. Routine monitoring and feedback of ordering data to program implementation staff throughout a time-limited vaccination program has the potential to facilitate timely identification of decreases in demand and enable corrective action as required.

Linking ordering patterns to programmatic activity retrospectively provides valuable insight into drivers of vaccine demand. The greatest amount of vaccine was ordered early in the program, which is a likely reflection of the ‘newness’ of the program. Programmatic activity related to this includes initial receipt of program information by parents/providers, providers preparing for anticipated parental demand, and a peak in the number of children eligible for vaccination. There is evidence to suggest that parental action is greatest in the first two months following commencement of a new vaccination program, thus increasing demand, which is also seen in this program [3]. Peaks in ordering were also seen later in the program, including at the start of the calendar year and when this survey was sent directly to providers. The second reminder letter from the CMO to parents of eligible but yet unvaccinated children did not correspond to an increase in vaccine orders, though an increase in vaccine uptake was observed in the month following this. As illustrated by the plateau in ordering in the second six months of the program, it is likely that providers had sufficient supply of 13vPCV from earlier in the program period to meet this demand.

To boost demand outside of the start and end months of a new time-limited vaccination program, future programs may benefit from re-distribution of program information and resources to providers, along with personalised reminders to parents of unvaccinated children. This would ideally be done mid-way through the program and it appears the most effective method for this is direct mail from the Government reinforced by local health staff (e.g. public health units).

### Notifying supplementary doses

The prominence of electronic notification to the ACIR, either directly from patient management software or via the ACIR website, is consistent with the increase in electronic notifications nationally [27]. Although this method has its advantages, such as less delay and reduced staff time, there is a need to routinely verify transmission to avoid errors which would lead to under-reporting [41]. Given the reliance on electronic notification systems it is imperative that they are updated with vaccine-specific codes prior to the introduction of a new vaccine on the NIP. However, as commercial companies provide patient management software, changes in the NIP are often not reflected in the software in a timely manner [42].

A definitive date for inclusion of a specific code for 13vPCV on the ACIR was not able to be determined, though survey respondents reported that there were delays in availability of this code that caused confusion for some providers. Most commonly respondents in this situation indicated they would use a generic code (‘other vaccine’) to notify the supplementary dose of 13vPCV. Due to limitations in the analysis of ACIR data, doses entered as ‘other vaccine’ are unable to be definitively included in coverage estimates from the ACIR, thus reducing reported coverage. However, explorative examination of the ‘other vaccine’ category on the ACIR indicates this would not fully account for the low reported coverage, which is most likely due to a combination of low uptake and under-reporting.

Under-reporting is a known limitation of the ACIR data, though the extent of this in recent years and for catch-up or supplementary dose vaccination programs is not currently known [43]. The difference between ACIR coverage (27%) and the estimated proportion of the eligible cohort for which 13vPCV supplementary doses were ordered (37%) was most likely due to under-reporting of doses administered or wasted. This difference is higher than available estimates of under-reporting for routine childhood vaccines (2–3%) [29,43,44]. This may be due to the perceived ‘optional’ nature of the supplementary dose and it not being linked to parental immunisation incentive payments. Another reason may be inaccurate recording on the ACIR due to the lack of a vaccine-specific code, thus limiting usefulness of electronic notification methods. A provider-level audit would be useful to more accurately determine the nature and extent of under-reporting for routine, catch-up and supplementary vaccination in Australia.

### Influences on uptake of the vaccine

The 13vPCV supplementary dose program provides protection for the six additional pneumococcal serotypes not covered in 7vPCV. As over 90% of children would have some protection against pneumococcal disease from their primary course of 7vPCV [45], there could be variability in the perception of parents and health professionals about the importance of protection from the additional serotypes [29]. The slightly higher coverage achieved in the 7vPCV catch-up program (12–23 month age group 40% vs 36%), which was the first pneumococcal vaccine universally introduced onto the NIP, provides some evidence to support this.

Uptake of the 13vPCV supplementary dose was higher in younger (aged 12–23 months) than older (aged 24–35 months) eligible children. This has also been observed in the United States (12–23 months of age 58% vs 24–59 months of age 32%) where reasons for this are thought to include parental perception of a greater disease risk in younger children and these children having more opportunities for vaccination as they visit health providers more frequently than older children [30]. This may also be the case in Australia where routine vaccination and health assessments primarily target children under 18 months and 3.5–4 years of age [46,47]. Therefore, offering the 13vPCV supplementary dose opportunistically when children presented for another health condition/vaccine and/or visited the immunisation provider with siblings would not have captured all eligible children.

Several comments from the provider survey illustrated circumstances where parents declined the 13vPCV supplementary dose as it was ‘another vaccine’ at 12 months of age. However, offering the 13vPCV supplementary dose at the 18-month NIP schedule point appeared effective, with nearly half (44%) of the children recorded on the ACIR as receiving a 13vPCV supplementary dose also received their 18-month NIP vaccine at the same time. The time and cost involved in making a separate appointment for a supplementary vaccine and having only one other vaccine administered concurrently were likely incentives for concurrent administration at 18 month NIP schedule point. Parental attitudes towards the 13vPCV supplementary dose program and reasons for non-vaccination were not in the scope of this evaluation, though would be beneficial to understand in order to further explain reasons for low uptake.

## Conclusion

Despite a high level of provider awareness, use of national resources to implement the program and reported high rates of electronic notification of administered doses to the ACIR, uptake of the 13vPCV supplementary dose was lower than for other comparable PCV catch-up vaccination programs. This was influenced by several factors, including; providers’ opportunistic approach to implementation, under-reporting of administered vaccines to the ACIR and parents’ passively or actively declining vaccines for their child.

This evaluation highlights several factors to consider when implementing future time-limited vaccination programs. Prior to vaccine distribution, it is important to ensure provision of program information to providers before patients. A follow-up targeted mail-out of key program information and resources mid-way through the program is likely to enhance demand. To facilitate notification, timely addition of vaccine-specific codes to reporting forms/software is important, as is educating providers about the importance of, and correct way to, report administered vaccines. Given the high proportion of eligible children vaccinated in the first six months of the program, consideration should be given to the duration of catch-up and supplementary dose programs, though this has to be weighed against cost-effectiveness and provision of ample opportunity for vaccination. Further evaluation of the effect of aligning supplementary (or catch up) vaccination programs with the NIP schedule is needed. This should include review of national data on timing of vaccine receipt as well as the effect on parents need to return for a separate appointment and prospects for opportunistic vaccination.
